# Systematic Review of Biopsychosocial Prognostic Factors for Return to Work After Acute Orthopedic Trauma: A 2020 Update

**DOI:** 10.3389/fresc.2021.791351

**Published:** 2022-02-04

**Authors:** Hong Phuoc Duong, Anne Garcia, Roger Hilfiker, Bertrand Léger, François Luthi

**Affiliations:** ^1^Department of Medical Research, Clinique Romande de Réadaptation, Sion, Switzerland; ^2^Department of Musculoskeletal Rehabilitation, Clinique Romande de Réadaptation, Sion, Switzerland; ^3^School of Health Sciences, HES-SO Valais-Wallis, Sion, Switzerland; ^4^Department of Physical Medicine and Rehabilitation, Orthopedic Hospital, Lausanne University Hospital, Lausanne, Switzerland

**Keywords:** return to work, orthopaedic trauma, injury, work disability, prognostic factors, biopsychosocial factors

## Abstract

**Objective:**

To provide updated evidence on prognostic factors for return to work (RTW) in the early and late phases after acute orthopedic trauma from a biopsychosocial perspective.

**Methods:**

A systematic review of articles indexed in the MEDLINE, CINAHL, and Embase databases between 2010 and 2020 was performed. The inclusion criteria were cohort studies of employed populations sustaining acute orthopedic trauma with follow-up data on RTW. Biopsychosocial prognostic factors for RTW must be reported in the multiple regression models and divided into early (≤ 6 months) and late phases (> 6 months) postinjury. Two reviewers performed study selection, assessed the risk of bias and quality using the Quality in Prognosis Studies (QUIPS) tool and the Newcastle–Ottawa Scale (NOS), and extracted data independently.

**Results:**

Thirty articles were included with a follow-up period of 1–58 months. Based on the QUIPS tool, 7 studies (23%) were considered to have a low risk of bias, and 21 studies (70%) were considered to have a moderate risk of bias. Based on the NOS, the quality was high in 87% of the included studies. The RTW rates ranged from 22% to 74% in the early phase and from 44% to 94% in the late phase. In the early phase, strong evidence was found for injury severity. In the late phase, strong evidence was found for age, injury severity, level of pain, self-efficacy, educational level, blue-collar work, and compensation status; moderate evidence was found for recovery expectations and physical workload. There was limited or inconsistent evidence for the other factors.

**Conclusion:**

Based on the levels of evidence, injury severity should be considered as one of the key barriers to RTW in the early and late phases postinjury. This finding underlines the need for serious injury prevention efforts. Our results also emphasize the multifaceted actions of the biopsychosocial model to facilitate RTW: promoting policies for older injured workers, improving access to medical and rehabilitation facilities, and adapting physical workload. Multiple other factors are likely important but require additional high-quality studies to assess their role in the RTW process.

## Introduction

Acute orthopedic trauma represents one of the most common injuries in workplace accidents, traffic accidents, and other types of accidents (1). It is also responsible for individual disability and loss of workdays and implies a substantial economic and societal burden (2, 3). According to the official statistical report of the Swiss accident insurance fund, among 850,000 accidents reported (8.7 million inhabitants) in 2018, orthopedic trauma accounts for 85% of all injuries (4). The mean direct and indirect costs due to orthopedic trauma in Switzerland between 2014 and 2018 were 3.96 billion euros (4). According to a systematic review of 204 studies in 2020, 13% of patients sustaining orthopedic trauma had lost employment at 1 year postinjury, and the mean number of days absent from work was 102 days (3).

Based on these data, return to work (RTW) after orthopedic trauma has become a key outcome for people of working age. RTW marks a return to financial independence for the individual and the end of compensation benefits for society. From an individual point of view, RTW is associated with better psychological well-being, self-esteem, and social connectedness (5). However, the definition and measurement of RTW outcome remain highly heterogeneous from study to study (6). A synthesis of the measurement of RTW outcome in the literature may help to clarify its operationalization.

Furthermore, the identification of prognostic factors for RTW remains the focus of many studies in the field. RTW seems to be influenced by different personal and environmental determinants due to its complex and multidimensional nature (7). Indeed, the usual biomedical model cannot fully explain the RTW process for patients with musculoskeletal disorders (8). The biomedical model, based on a dualistic mind-body viewpoint, fails to take into account psychological, social, and health system aspects. These psychosocial factors were, however, widely recognized as having an independent influence on RTW (7–9). Another limitation of the biomedical model is its inability to explain the interaction between injury severity and other psychosocial factors in predicting the long-term outcome. The biopsychosocial (BPS) model developed by George Engel in 1977 (10), based on a holistic approach, might overcome the limitations of the traditional biomedical framework for predicting multidimensional outcomes such as RTW (11). The underlying assumptions of the BPS model rely on the complex and non-linear interactions between the biological, psychological, and social determinants that affect disease outcomes (12). Based on this model, the RTW process depends not only on biomedical characteristics but also on personal and environmental factors (workplace, healthcare system, compensation policy) (13). Early identification of BPS factors associated with RTW is of importance to help in developing effective interventions to prevent work disability and subsequently in reducing the personal and societal burden of orthopedic trauma.

In 2010, the prognostic factors involved in the RTW process in patients with orthopedic trauma were presented in a systematic review by Clay et al. (9). At that time, the literature was scarce, and the quality of the studies was limited. According to an earlier review (9), two factors of strong evidence identified were educational levels and blue-collar work, whereas three factors of moderate evidence were self-efficacy, injury severity, and compensation status. Over the past 10 years, the emergence of new evidence has raised the need to perform an updated review on this topic.

In this systematic review, we updated the latest evidence on prognostic factors for RTW in employed populations sustaining acute orthopedic trauma by reviewing studies indexed in three large databases between 2010 and 2020. Prognostic factors were evaluated using a two-tiered strategy (significant and non-significant effects) and classified into the early or late phase postinjury using a 6-month cutoff. This cutoff point was used when assuming that some orthopedic injuries (for example, fractures) might take up to 6 months to recover (3) and that the influence of some prognostic factors on RTW might vary in a timely fashion (14).

## Methods

### Protocol and Registration

The review protocol was performed following the “Preferred Reporting Items for Systematic reviews and Meta-Analysis” (PRISMA) recommendations (15) (see Appendix PRISMA 2020 checklist). The research protocol was registered in PROSPERO (CRD42017074234).

### Eligibility Criteria

This literature review was extended to articles written in English, French, and German. The articles had to be available in full text and as published articles (conference papers/abstracts were excluded).

We included studies published between January 1, 2010 and December 31, 2020 and fulfilled the following criteria:

-**Study designs**: prospective or retrospective studies with longitudinal data on RTW.

-**Participants**: studies on individuals with acute orthopedic trauma only or orthopedic trauma represented a minimum of 75% of the sample and were employed at the time of injury. Acute orthopedic trauma is defined as any injury (strains/sprains, contusions, dislocations, and fractures) to the musculoskeletal system due to an unintentional accident.

-**Outcome measures**: RTW was defined as the return to the preinjury or modified job (fully or partially) or a period of time off work or not being prevented from working at a certain follow-up measurement or RTW sustained for a long period.

-**Prognostic factors**: Biological (age, gender, level of pain, etc.), psychological (depression, anxiety, etc.), and social factors (education, occupation, work-related environment, etc.) were eligible. Considering the multidimensional nature of work incapacity, the factors must be reported in the multiple regression models. We did not include the unadjusted effects of prognostic factors in the analysis because their effects may completely disappear after adjustment and are therefore relatively uninformative. Adjusted effects of prognostic factors were extracted for data analysis and were divided into the early phase (≤ 6 months from injury) and the late phase (> 6 months from injury).

Exclusion criteria were as follows: whiplash, brain injury, medullar injury, or injuries resulting from occupational repeated trauma; no precision about the percentage of orthopedic trauma, sample size lower than 80 individuals (to rule out findings with low statistical power), soldiers or military population; and retrospective without follow-up data on RTW or cross-sectional studies (to limit recall bias).

### Search Strategy

We performed a literature search in the MEDLINE, CINAHL, and Embase databases using keywords covering three areas: (a) RTW or work absence or work disability or sick leave or time off work; (b) orthopedic trauma or injury or fracture; and (c) prognostic or prognosis or risk factors or outcome. Additional manual searching of reference lists of all included studies was performed. The terms within each area were combined with an OR Boolean operator, and then, the three areas were combined with an AND Boolean operator. The search results were uploaded to the Endnote program. The duplicates were removed. Two of the authors (AG and RH) made the first selection of articles based on the abstracts from 2010 to 2017 and (AG and HPD) from 2017 to 2020. Next, the full-text articles were obtained by the researchers, and they included the relevant articles independently according to the predefined criteria and then compared their choices. The final decision of inclusion was based on consensus. The last senior author (FL) made the final decision if no consensus was reached.

### Data Extraction and Analysis

In accordance with the Critical Appraisal and Data Extraction for Systematic Reviews of Prediction Modeling Studies (CHARMS) checklist (16) for data extraction, the following elements were extracted from the included studies: first author and year, country, setting, nature of the trauma, type of study, definition of the outcome, sample size in the final multiple regression model, duration of follow-up, percentage lost to follow-up, predictor measurements, and RTW rate. The data from selected studies were extracted independently by the aforementioned reviewers. Significant barriers and facilitators for RTW and also non-significant factors were reported with their statistical values. Odds ratios, relative rate ratios, hazard ratios, or regression coefficients with 95% confidence intervals were extracted from the final multivariable models with imputation if available. Finally, we classified the factors according to the BPS categories and their implications in the early or late phase postinjury. We planned to conduct meta-analyses where this was appropriate; otherwise, we summarized narratively.

### Risk of Bias and Quality Assessment

For studies of prognostic factors, the Quality in Prognosis Studies (QUIPS) tool was used to assess the risk of bias (17). The QUIPS tool has six domains: (1) study participants, (2) study attrition, (3) prognostic factor measurement, (4) outcome measurement, (5) study confounding, (6) statistical analysis and reporting. Each domain includes three to seven items that are judged separately with the response “yes,” “partial,” “no,” or “unsure.” Based on the ratings of the included items, the risk of bias within each domain is expressed as high, moderate, or low. A study was classified as having a low risk of bias when all domains were rated as having a low risk of bias or up to one moderate risk of bias. A study was classified as having a high risk of bias if two or more of the domains were rated as having a high risk of bias. All studies in between were classified as having a moderate risk of bias.

For studies presenting a prognostic model, prediction model risk of bias assessment tool (PROBAST) was used to assess the risk of bias (18). The PROBAST consists of 20 signaling questions grouped into four domains: participant selection, predictors, outcome, and analysis (18). All signaling questions answered as “yes” indicate an absence of bias. Any signaling question answered as “no” or “probably no” flags the potential for bias; assessors would need to use their judgment to determine whether the domain should be rated as high, low, or unclear risk of bias.

The quality of the included studies was assessed by the Newcastle–Ottawa Scale (NOS) (19). The NOS consists of three categories of parameters (selection, comparability, and outcome) with a total of 9 points. A study of a total of NOS scores > 6 points, 5 or 6 points, and ≤ 4 points was rated as having high, medium, and low quality, respectively.

Two of the authors jointly (AG and HPD) assessed the risk of bias and quality of the articles. Disagreements were resolved by consulting the last author to achieve consensus.

### Levels of Evidence

To be retained, the prognostic factors must have been measured in the multiple regression model. To compare with the previous review on RTW (9), the levels of evidence were determined by using a rating system similar to that used by Scholten-Peeters (20). According to this system, there were four levels of evidence: strong/moderate/limited/inconsistent. **Strong evidence**: consistent findings were found in at least two high-quality cohorts with a low risk of bias. **Moderate evidence**: consistent findings were found in one high-quality cohort with a low risk of bias and one or more cohorts with a moderate or high risk of bias regardless of the level of quality. **Limited evidence**: consistent findings in one regardless of the level of risk of bias or more cohorts with moderate or high risk of bias. **Inconsistent evidence:** contradictory findings were found irrespective of study quality.

## Results

### Characteristics of Included Studies

**Figure 1:**
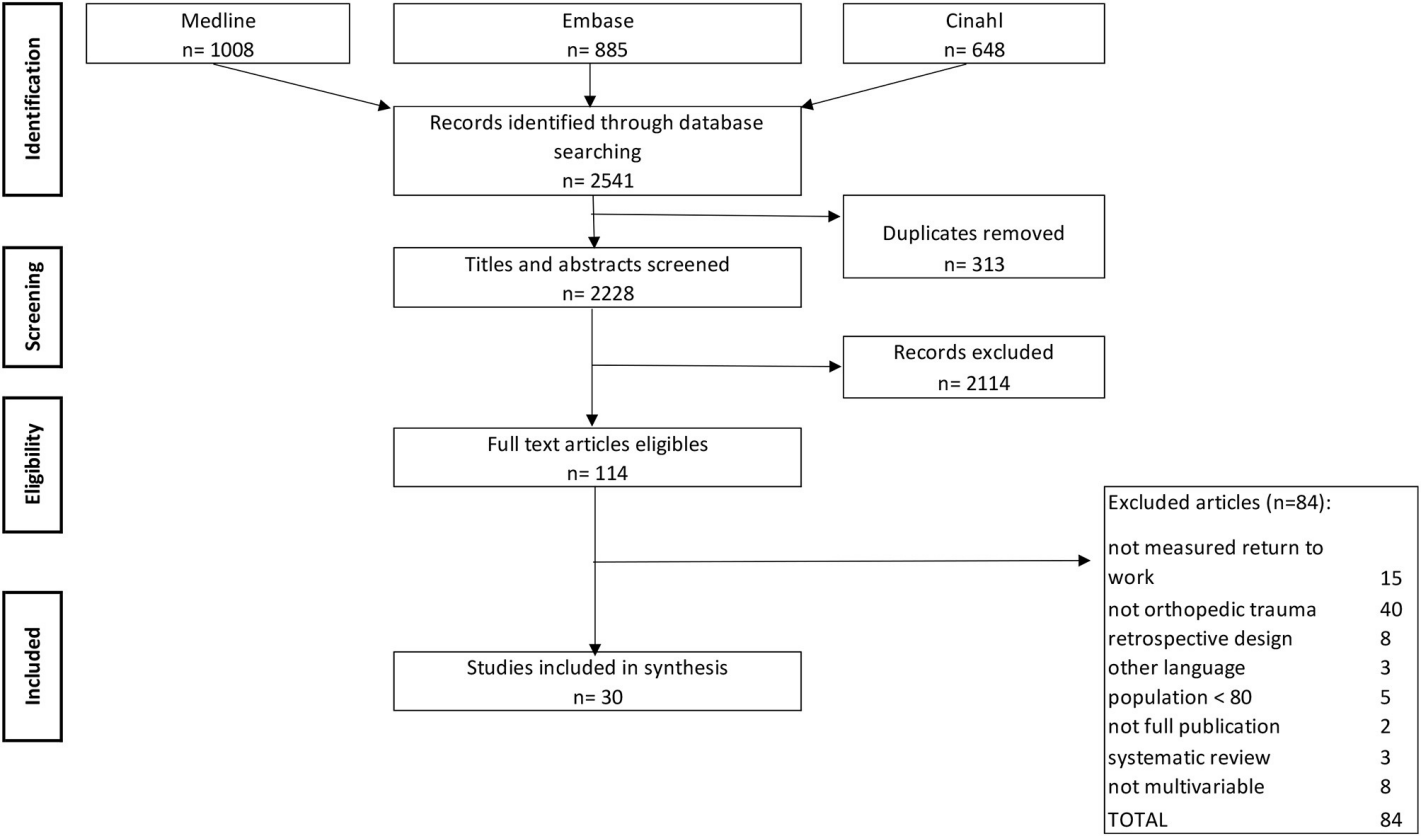
Flow diagram for selection of studies for this systematic review.

The flow diagram is presented in Figure 1, and reasons for exclusions at each stage are provided. The initial search yield 2,541 articles. After removing duplicates and screening titles and abstracts, 114 articles were retained for further assessment of eligibility. After reading these full-text articles, 30 articles met the inclusion criteria.

The characteristics of the selected studies are summarized in Table 1. Twenty-five studies were prospective (21–37, 41–47, 50), and five were retrospective but included longitudinal data on RTW (38–40, 48, 49). The follow-up time ranged from 1 to 58 months postinjury. The RTW rates ranged from 22 to 74% in the early phase postinjury (22, 28, 35, 36, 41, 43, 44) and from 44 to 94% (21–32, 34, 37, 38, 40, 42, 43, 45, 46, 48–50) in the late phase.

**Table 1:** Characteristics of included studies (*n* = 30).

**References**	**Country**	**Setting**	**Nature of injuries**	**Study design**	**Outcome definition**	**Follow-up (months)**	**Predictors measures**	**Lost to follow-up**	**Number in multiple regression analysis**	RTW rates (months)^*^
Amick et al. (21)	Canada	Regional database	Back + upper extremities trauma	Prospective	RTW status (any type of work) yes/no	6, 12	1 month postinjury	31%	577	74% (6) 76% (12)
Ballabeni et al. (22)	Switzerland	Clinic	Orthopedic trauma	Prospective	Return to any occupation	3, 12, 24	At entry and discharge	45%	291	37% (3), 45% (12), 56% (24)
Busse et al. (23)	Canada	Multi-center	Tibial shaft fracture	Prospective	NA	12	6 weeks after injury	29%	186	64% (12)
Clay et al. (24) Clay et al. (25) Clay et al. (26)	Australia Australia Australia	Victorian hospital dataset	Acute orthopedic injuries	Prospective	RTW to full duties or modified work	6	Preinjury and 2 week postinjury	10%	168	44% return to full duties (6), 56% return to modified work (6)
Clay et al. (27)	Australia	Multicenter	Orthopedic trauma >75%	Prospective	Time until the first RTW on either preinjury or reduced hours	12	1–6 week postinjury	53%	186	81% (12)
Dinh et al. (28)	Australia	Hospital	Trauma patients	Prospective	NA	3, 6 postdischarge	At baseline	20%	179	74% (3), 76% (6)
Eisele et al. (29)	Germany	9 centers	Hand trauma	Prospective	Time between injury and RTW	1.5, 3, 6	Fist admission		231	77% (6)
Ekegren et al. (30)	Australia	VOTOR registry	Hip fracture patient	Prospective	Paid employment, same workplace, same role, or others	12	At baseline	22%	291	65% (12)
Gabbe et al. (31)	Australia	VOTOR registry	Orthopedic trauma	Prospective	RTW yes/no status	12	At admission	13%	953	70% (12)
Hou et al. (32)	Taiwan	Hospital	Limb trauma injury	Prospective	“without RTW” or “RTW”	1, 3, 6, 12, 18, 24	NA	0%	1,124	75% (24)
Hou et al. (33)	Taiwan	Hospital	Traumatic limb injury	Prospective	Same or other job, same workplace, or other workplace	1, 6, 24	NA	NA	804	22% (1) 50% (6)
Iakova et al. (34)	Switzerland	Clinic	Orthopedic trauma	Prospective	Has a job or not (binary response)	24	At admission	34%	1,207	58% (24)
Izadi et al. (35)	Iran	Hospital	Hand trauma	Prospective	Time to RTW after surgery	3	1–8 weeks after surgery	NA	280	46% (3)
Kendrick et al. (36)	The United Kingdom	UK Burden of Injury Study	Orthopedic trauma >80%	Prospective	Not being prevented from working for any days in the last 4 weeks	1, 4	1–4 weeks after injury	50%	664	73% (4)
Kendrick et al. (37)	The United Kingdom	Hospital	Upper/lower extremities	Prospective	Full or part- time paid employment and not being prevented from working	2, 4, 12	1 month postinjury	24%	273	67% (12)
Kimmel et al. (38)	Australia	VOTOR registry	Isolated lower limb fracture	Assessed prospective	RTW status Yes/no	12	Preinjury	15%	6,775	84% (12)
Kirkeby et al. (39)	Denmark	Hospital	Wrist trauma suspicion of scaphoid fracture	Retrospective with follow-up	Completion of a period of four consecutive weeks on labor market	58	At admission	NA	125	NA
Kong et al. (40)	China	Hospital	Work-related injuries	Retrospective with longitudinal data	RTW sustained for 3 months	8	At admission	25%	335	78% (8)
Lilley et al. (41)	New Zealand	ACC claim register	Workers with orthopedic trauma >75%	Prospective	Return to any form of work	3	3 months postinjury	1%	2,250	73% (3)
Luthi et al. (42)	Switzerland	Clinic	Orthopedic trauma	Prospective	Return to same or modified job	24	At admission	27%	819	50% (24)
Marom et al. (43)	Israel	Clinic	Hand trauma	Prospective	RTW status: Yes/no	3,6,9,12	At admission	1%	178	32% (3) 65% (6) 74% (9) 75% (12)
Marom et al. (44)	Israel	Clinic	Hand trauma	Prospective	RTW status: yes/no	3	At admission	0%	178	37% (3)
Murgatroyd et al. (45)	Australia	Hospital	Orthopedic trauma	Prospective	full/modified duties, time from injury to work	6, 12, 24	2 weeks postinjury	44%	182	65% (6), 73% (12), 81% (24)
Neutel et al. (46)	Netherlands	Hospital	Hand/wrist trauma	Prospective	Time to resume work fully	10	2 weeks after the trauma	13%	354	94% (10)
Roesler et al. (47)	Australia	Hand therapy clinic	Traumatic hand injury	Prospective follow-up	NA	3	4 weeks after injury	NA	150	NA
Tay et al. (48)	Australia	VOTOR	Femoral and tibial shaft fractures	Retrospective but longitudinal data	Return to any form of work	6, 12	At admission	25–35%	489	50% (6) 67% (12)
Vuistiner et al. (49)	Switzerland	Clinic	Orthopedic trauma	Retrospective but follow-up	Number of days paid for work disability	48	At admission	26%	807	50% (12)
Yang et al. (50)	Australia	VOTOR	Traumatic vertebral fracture	Prospective	RTW status Yes/no	12	NA	23%	264	77% (12)

*RTW return to work NA: not available*.

* *RTW rates at a specific month*.

Return to work was defined as a completion of a period of four consecutive weeks on the labor market in one study (39), as a sustained outcome for 3 months in one study (40), as a return to any form of work (the same or modified work, full time or part time) in 18 studies (21, 22, 24–27, 30, 32, 33, 37, 41–46, 48, 49), or as not being prevented from working for any days in the last 4 weeks in one study (36). Nine studies did not provide the definition (23, 28, 29, 31, 34, 35, 38, 47, 50). Concerning its measurement, RTW was reported as a binary outcome (yes/no status) in 23 studies (21–26, 28, 30–34, 36–38, 40–44, 47, 48, 50), as time from injury to RTW in 6 studies (27, 29, 35, 39, 45, 46), or as the number of days paid for work disability in one study (49).

The rates of loss to follow-up were reported in 25 studies (21–28, 30–32, 34, 36–38, 40–46, 48–50), ranging from 1 to 53%. The studies were conducted in 12 different countries of high or upper-middle income (Australia, Switzerland, Taiwan, Canada, Israel, the United Kingdom, Germany, the Netherlands, Denmark, New Zealand, China, and Iran). Of note, most European countries were the part of universal health coverage, whereas in many other countries (Australia and the United Kingdom), private companies were involved in compensation with different sick pay schemes.

### Risk of Bias and Quality Assessment

**Figure 2:**
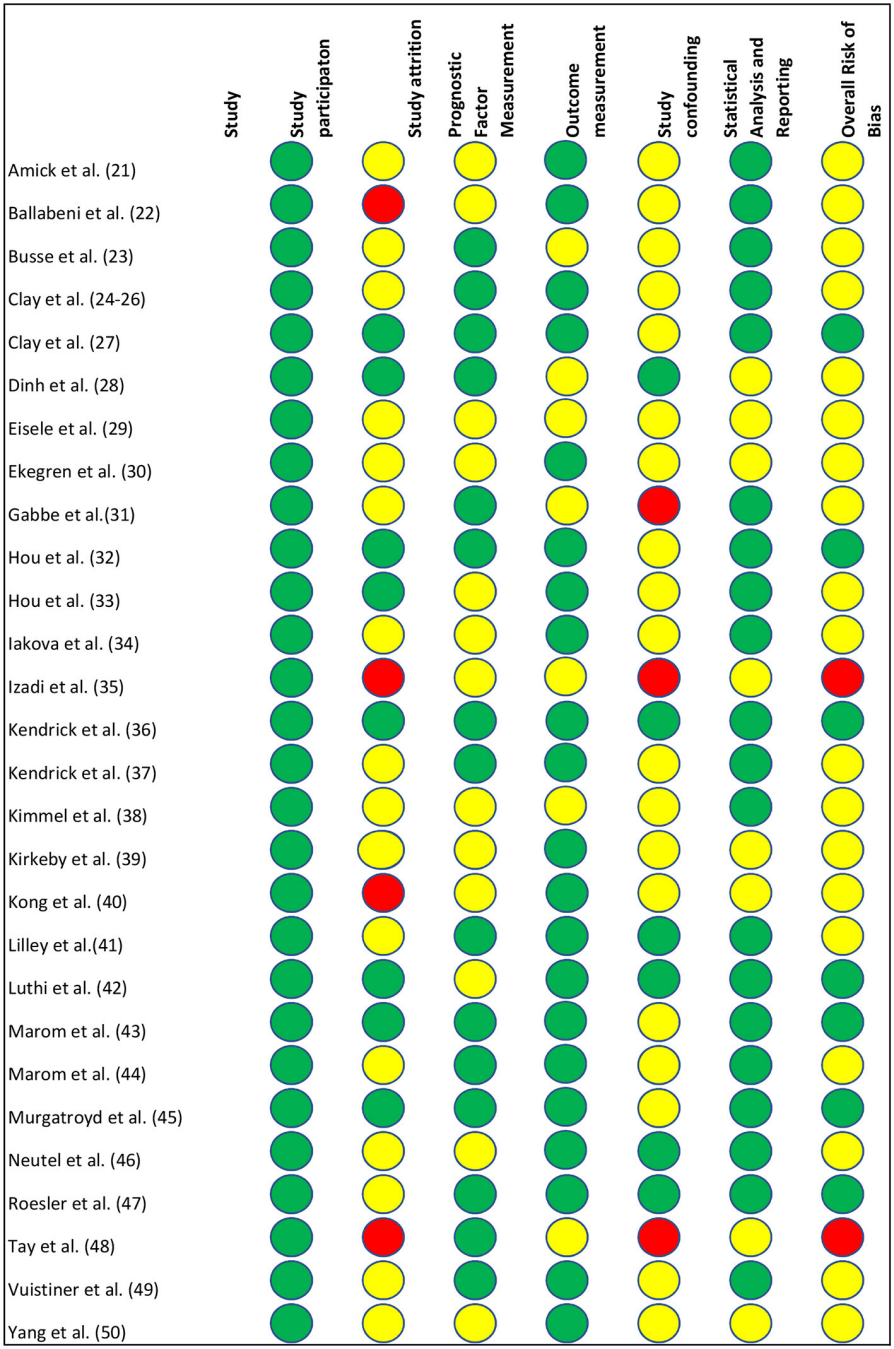
Risk of bias according to the Quality in Prognosis Studies (QUIPS) tool. Red circle = high risk of bias, yellow circle = moderate risk of bias, green circle = low risk of bias.

A majority of the included studies (97%) reported the statistical estimates of an array of predictors of RTW outcome, except one (42) that presented a prognostic model for RTW with external validation. Therefore, we used the QUIPS tool, which was developed to assess the risk of bias in predicting factor studies for all included studies. Figure 2 represents the overall risk of bias of the included studies using the QUIPS tool and also the ratings of each domain. The agreement between the two reviewers for the QUIPS tool was 87% (26 out of 30 studies). Disagreement was found in the rating of study attrition and confounding factors. Seven studies (23%) were rated as having a low risk of bias (27, 32, 36, 42, 43, 45, 47), 2 studies (7%) as having a high risk of bias (35, 48), and the 21 remaining studies (70%) as having a moderate risk (21–26, 28–31, 33, 34, 37–41, 44, 46, 49, 50).

For the study presenting a prognostic model for RTW (42), apart from the QUIPS, we also used PROBAST to assess the risk of bias. The risk of bias in the four domains (participants, predictors, outcome, and analysis) based on PROBAST was low; therefore, an overall low risk of bias was drawn.

The quality of the included studies was also assessed using the NOS (Table 2). Twenty-six studies (87%) had a total NOS score between 7 and 8 points and therefore were rated as high quality. The remaining studies were rated as having medium quality.

**Table 2:** Quality assessment of included study based on the Newcastle-Ottawa Scale.

**References**	**Selection (score)**			**Comparability (score)**			**Outcome (score)**		
	**Representativeness of the exposed cohort**	**Selection of the non-exposed cohort**	**Ascertainment of exposure**	**Outcome of interest was not present at start of study**	**Based on the design or analysis**	**Assessment of outcome**	**Follow-up long enough for outcomes to occur**	**Adequacy of follow-up of cohorts**	**Total score**
Amick et al. (21)	1	0	1	1	2	1	1	0	7
Ballabeni et al. (22)	1	0	1	1	2	1	1	0	7
Busse et al. (23)	1	0	1	1	2	1	1	0	7
Clay et al. (24–26)	1	0	1	1	2	1	1	0	7
Clay et al. (27)	1	1	1	1	2	1	1	0	8
Dinh et al. (28)	1	0	1	1	2	1	1	0	7
Eisele et al. (29)	1	0	1	1	2	1	1	0	7
Ekegren et al. (30)	1	1	1	1	1	1	1	0	7
Gabbe et al. (31)	1	0	1	1	1	1	1	0	6
Hou et al. (32)	1	0	1	1	2	1	1	1	8
Hou et al. (33)	1	1	1	1	2	1	1	0	8
Iakova et al. (34)	1	0	1	1	2	1	1	1	8
Izadi et al. (35)	1	0	1	0	1	1	1	0	5
Kendrick et al. (36)	1	1	1	1	2	1	1	0	8
Kendrick et al. (37)	1	0	1	1	2	1	1	0	7
Kimmel et al. (38)	1	1	1	1	1	1	1	0	7
Kirkeby et al. (39)	1	0	1	1	1	1	1	0	6
Kong et al. (40)	1	0	1	1	2	1	1	0	7
Lilley et al. (41)	1	0	1	1	2	1	1	0	7
Luthi et al. (42)	1	1	1	1	2	1	1	0	8
Marom et al. (43)	1	0	1	1	2	1	1	1	8
Marom et al. (44)	1	0	1	1	1	1	1	1	7
Murgatroyd et al. (45)	1	0	1	1	2	1	1	1	8
Neutel et al. (46)	1	0	1	1	2	1	1	1	8
Roesler et al. (47)	1	0	1	1	2	1	1	0	7
Tay et al. (48)	1	0	1	1	1	1	1	0	6
Vuistiner et al. (49)	1	0	1	1	2	1	1	0	7
Yang et al. (50)	1	0	1	1	2	1	1	1	8

### Prognostic Factors for RTW

The biopsychosocial factors that showed a significant association with RTW in the early and late stages are presented in Tables 3, 4, respectively.

**Table 3:** Barriers and facilitators for RTW in the early phase (≤ 6 months) postinjury.

**Factors**	**Authors**	**Categories of interest**	Barriers for RTW^*^	Facilitators for RTW^*^	**Statistical reported**	**Levels of evidence**
**Biological factors (*****n*** **=** **7)**						
Injury severity	Izadi et al. (35)	Modified hand injury severity scale	11.45 (6.88–16.02)		Coef (95% CI)	Strong
	**Roesler et al**. **(****47****)**	**Modified hand injury severity scale**	**1.66**		**OR**	
	**Kendrick et al**. **(****36****)**	**Abbreviated injury scale**	**0.79 (0.68–0.92)**		**RR (95% CI)**	
	Lilley et al. (41)	Hospital admission for injury	2.10 (1.66–2.64)		OR (95% CI)	
	Clay et al. (24)	Isolated vs. Mutiple injury		2.80 (1.10–6.97)	OR (95% CI)	
	Clay et al. (25)	Injury Severity Scores > 9	0.63 (0.39–0.99)		RRR (95% CI)	
Disability post-injury	Marom et al. (44)	WHO-DAS II	0.96 (0.93–0.99)		OR (95% CI)	Limited
	Izadi et al. (35)	Work DASH	0.60 (0.32–0.88)		Coef (95% CI)	
**Other factors**						
	**Kendrick et al**. **(****36****)**	**Male vs. female**		**1.94 (1.34–2.82)**	**RR (95% CI)**	Limited
	Lilley et al. (41)	BMI (Obese vs. normal)	1.48 (1.13–1.94)		OR (95% CI)	Limited
	Clay et al. (25)	Increasing age	0.98 (0.96–0.99)		RRR (95% CI)	Limited
	Clay et al. (25)	McGill Pain Questionnaire	0.47 (0.27–0.82)		RRR (95% CI)	Limited
	Clay et al. (25)	Initial need for surgery	0.61 (0.39–0.96)		RRR (95% CI)	Limited
**Psychological factors (*****n*** **=** **6)**						
Other factors	**Roesler et al**. **(****47****)**	**Positive and negative affect scale**	**1.14**		**OR**	Limited
	**Roesler et al**. **(****47****)**	**Health locus of control**	**5.11**		**OR**	Limited
	Lilley et al. (41)	Prior depressive episode	1.27 (1.02–1.59)		OR (95% CI)	Limited
	Hou et al. (33)	Psychological subscale	1.15 (1.01–1.30)		OR (95% CI)	Limited
	Clay et al. (25)	Negative pain attitudes	0.49 (0.31–0.77)		RRR (95% CI)	Limited
	Clay et al. (26)	Recovery belief, strong		16.73 (3.59–77.88)	OR (95% CI)	Limited
**Social factors (*****n*** **=** **20)**						
Education	Marom et al. (44)	Education ≥ 12 years		3.44 (1.35–8.73)	OR (95% CI)	Limited
	Kong et al. (40)	Secondary vs. primary school		2.5 (1.3–4.9)	HR(95% CI)	
	Clay et al. (26)	University		6.27 (1.72–22.9)	OR (95% CI)	
Blue-collar work	Lilley et al. (41)	Blue-collar vs. white-collar	1.52 (1.14–2.02)		OR (95% CI)	Limited
	Clay et al. (25)	Blue-collar vs. white-collar	0.52 (0.32–0.84)		RRR (95% CI)	
Other factors	Marom et al. (44)	Use of legal counsel (yes)	0.45 (0.20–1.00)		OR (95% CI)	Limited
	Izadi et al. (35)	Smoker	7.91 (1.41–14.41)		Coef (95% CI)	Limited
	Izadi et al. (35)	Job title			Coef (95% CI)	Limited
	**Roesler et al**. **(****47****)**	**Number of people in houshold**		**0.015**	**OR**	Limited
	**Kendrick et al**. **(****36****)**	**Injured at work (yes vs. no)**	**0.49 (0.27–0.87)**		**RR (95% CI)**	Limited
	Lilley et al. (41)	Temporary vs. permanent contract	1.89 (1.27–2.81)		OR (95% CI)	Limited
	**Kendrick et al**. **(****36****)**	**Self employed vs. paid employment**		**1.15 (1.03–1.30)**	**RR (95% CI)**	Limited
	Lilley et al. (41)	6–7 vs. 5 days	1.54 (1.21–1.96)		OR (95% CI)	Limited
	Lilley et al. (41)	Physial work	1.93 (1.38–2.72)		OR (95% CI)	Limited
	**Kendrick et al**. **(****36****)**	**Living in deprived areas**	**0.59 (0.40–0.85)**		**RR (95% CI)**	Limited
	Lilley et al. (41)	Income (<30.000 vs. >5000 USD)	1.81 (1.33–2.48)		OR (95% CI)	Limited
	Lilley et al. (41)	Financial security (insecure)	1.55 (1.22–1.96)		OR (95% CI)	Limited
	Lilley et al. (41)	Exercise (7 days vs. ≤ 4 days)		0.67 (0.54–0.83)	OR (95% CI)	Limited
	Kong et al. (40)	Family's attituted to RTW		4.0 (1.4–11)	HR (95% CI)	Limited
	Kong et al. (40)	Perception of social support		1.9 (1.2–3.0)	HR (95% CI)	Limited
	Kong et al. (40)	Computer skill training (yes)		1.5 (1.1–2.1)	HR (95% CI)	Limited
	Clay et al. (25)	Social functioning (SF36)		1.89 (1.17–3.07)	RRR (95% CI)	Limited
	Clay et al. (26)	Received compensation	0.23 (0.09–0.61)		OR (95% CI)	Limited

*RTW, Return to work; OR, odds ratio; RR, relative risk; Coef, coefficient; HR, Hazard ratio; RRR, relative rate ratio; CI, confidence interval*.

* *Barriers or facilitators for RTW were reported in positive or negative direction depending on the definition of outcome, or the categories of interest, or the statistical reported*.

*Studies with a low risk of bias and high-quality were bolded*.

**Table 4A:** Barriers and facilitators for RTW in the late phase (> 6 months) postinjury (part 1).

**Factors**	**Authors**	**Categories of interest**	Barriers for RTW^*^	Facilitators for RTW^*^	**Statistical reported**	**Levels of evidence**
**Biological factors (*****n*** **=** **20)**						
Age	Ekegren et al. (30)	55–64 vs. 16–24	0.11 (0.03–0.40)		OR (95% CI)	Strong
	**Hou et al**. **(****32****)**	**Age** **>65 vs**. ** <65**	**0.28 (*****p*** **<** **0.001)**		**coef**	
	**Luthi et al**. **(****42****)**	**Inscreasing age, per 10 years**	**1.19 (1.07–1.34)**		**OR (95% CI)**	
	Busse et al. (23)	Increasing age, per 10 years	0.74 (0.33–1.69)		OR (95% CI)	
	Hou et al. (33)	Increasing age	1.04 (1.01–1.06)		OR (95% CI)	
	**Clay et al**. **(****27****)**	**Increasing age**	**0.97 (0.96–0.99)**		**HR (95% CI)**	
Injury severity	Neutel et al. (46)	presence of complication	1.88 (1.04–3.42)		HR (95% CI)	Strong
	Ekegren et al. (30)	Isolated vs. non-isolated injury	0.31 (0.15–0.64)		OR (95% CI)	
	**Murgatroyd et al**. **(****45****)**	**New injury severity score**	**0.54 (0.35–0.82)**		**HR (95% CI)**	
	Dinh et al. (28)	Injury severity score	0.98 (0.97–0.99)		OR (95% CI)	
	Busse et al. (23)	Multi vs. no multi trauma	0.44 (0.18–0.74)		OR (95% CI)	
	Kong et al. (40)	Least serious to serious		3.5 (2.0–6.0)	HR (95% CI)	
	Hou et al. (33)	Hospitalization days	1.18 (1.1–0.25)		OR (95% CI)	
	**Clay et al**. **(****27****)**	**Severe vs. minor/moderate**	**0.41 (0.26–0.66)**		**HR (95% CI)**	
Pain level	**Marom et al**. **(****43****)**	**Visual analog scale**	**0.91 (0.85–0.98**		**HR (95% CI)**	Strong
	Vuistiner et al. (49)	Brief pain inventory	0.67 (0.59–0.76)		HR (95% CI)	
	Vuistiner et al. (49)	Pain decrease		1.46 (1.3–1.64)	HR (95% CI)	
	**Clay et al**. **(****27****)**	**Symptomatic pain**	**0.47 (0.30–0.75)**		**HR (95% CI)**	
	Iakova et al. (34)	Visual analog scale	0.59 (0.59–0.59)		OR (95% CI)	
	Iakova et al. (34)	Pain decrease		1.69 (1.47–2.04)	OR (95% CI)	
Gender	Neutel et al. (46)	Female vs. Male	1.61 (1.22–2.12)		**HR (95% CI)**	Inconsistent
	**Clay** et al. **(****27****)**	**Female vs. Male**		**2.05 (1.22–3.46)**	**HR (95% CI)**	
	Vuistiner et al. (49)	Female vs. Male		1.29 (1.14–1.47)	HR (95% CI)	
Other factors	Kimmel et al. (38)	Discharge to rehabilitation		0.34 (0.26–0.46)	OR (95% CI)	Limited
	**Marom et al**. **(****43****)**	**Post-injury disability**	**0.98 (0.97–0.99)**		**HR(95% CI)**	Limited
	Neutel et al. (46)	Diagnosis other than wrist pain	2.48 (1.63–3.76)		HR (95% CI)	Limited
	Eisele et al. (29)	Joint functions		1.63(1.17–2.26)	HR (95% CI)	Limited
	Eisele et al. (29)	Sensory funtions		2.33 (1.45–3.74)	HR (95% CI)	Limited
	Kirkeby et al. (39)	MRI findings (yes)	0.48 (0.29–0.80)		HR (95% CI)	Limited
	Kendrick et al. (37)	Increased hospital stay	0.91 (0.86–0.96)		OR (95% CI)	Limited
	Ekegren et al. (30)	Pre–injury disability	0.21 (0.07–0.60)		OR (95% CI)	Limited
	**Murgatroyd et al**. **(****45****)**	**Never smoked**		**1.54 (1.04–2.23)**	**HR (95% CI)**	Limited
	**Murgatroyd et al**. **(****45****)**	**Pre–injury health status**	**0.36(0.14–0.91)**		**HR (95% CI)**	Limited
	**Hou et al**. **(****32****)**	**lower limbs vs. upper limbs**	**0.29 (*****p*** **<** **0.001)**		**coef**	Limited
	Vuistiner et al. (49)	EuroQol−5D	1.16 (1.13–1.19)		HR (95% CI)	Limited
	Tay et al. (48)	Delayed union in fracture limb	0.76 (0.57–0.94)		RR (95% CI)	Limited
	Busse et al. (23)	Open vs. close fracture	0.36 (0.18–0.74)		OR (95% CI)	Limited
	Hou et al. (33)	Lower limb vs. upper limb	3.63 (2.00–6.60)		OR (95% CI)	Limited
	Yang et al. (50)	Burst fracture vs. no burst	0.46 (0.22–0.97)		OR (95% CI)	Limited
	Yang et al. (50)	Radius vs. no radius fracture	0.12 (0.03–0.43)		OR (95% CI)	Limited

*RTW, Return to work; OR, odds ratio; RR, relative risk; Coef, coefficient; HR, Hazard ratio; CI, confidence interval*.

* *Barriers or facilitators for RTW were reported in positive or negative direction depending on the definition of outcome, the categories of interest, and the statistical reported*.

*Studies with a low risk of bias and high-quality were bolded*.

**Table 4B:** Barriers and facilitators for RTW in the late phase (> 6 months) postinjury (part 2).

**Factors**	**Authors**	**Categorgies of interest**	Barriers for RTW^*^	Facilitators for RTW^*^	**Statistical reported**	**Levels of evidence**
**Psychological factors (*****n*** **=** **8)**						
Self efficacy	**Marom et al**. **(****43****)**	**Decreased level**	**1.34 (1.10–1.64)**		**HR (95% CI)**	Strong
	**Hou et al**. **(****32****)**	**High vs. no chance**		**0.352 (*****p*** **<** **0.001)**	**coef**	
	Hou et al. (33)	High vs. no chance		0.20 (0.09–0.47)	OR (95% CI)	
Recovery expectation	**Murgatroyd et al**. **(****45****)**	**Recovery expectations**		**2.09 (1.50–2.94)**	**HR (95% CI)**	Moderate
	Vuistiner et al. (49)	Positive expectation		1.50 (1.32–1.70)	HR (95% CI)	
Depressive/Anxiety	Kendrick et al. (37)	Depression	0.87 (0.79–0.95)		OR (95% CI)	Limited
	Hou et al. (33)	Depressive symptoms	1.11 (1.03–1.20)		OR (95% CI)	
Perception of injury	Vuistiner et al. (49)	High perceived	0.72 (0.61–0.85)		HR (95% CI)	Limited
	Iakova et al. (34)	Low perceived		1.08 (1.03–1.14)	OR (95% CI)	
Other factors (*n* = 4)	**Marom** et al. **(****43****)**	**Intrusion thoughts**	**0.70 (0.57–0.86)**		**HR (95% CI)**	Limited
	Busse et al. (23)	illness beliefs	0.60 (0.50–0.73)		OR (95% CI)	Limited
	Iakova et al. (34)	lower avoidance		0.69 (0.61–0.79)	OR (95% CI)	Limited
	**Clay et al**. **(****27****)**	**Mental health (Poor vs. good)**	**0.57 (0.35–0.91)**		**HR (95% CI)**	Limited
**Social factors (** ***n** **=** **18*** **)**						
Blue–collar work	Neutel et al. (46)	Blue–collar vs. white–collar	2.52 (1.89–3.37)		HR (95% CI)	Strong
	**Murgatroyd et al**. **(****45****)**	**Manual workers vs. white–collar**	**0.53 (0.43–0.83)**		**HR (95% CI)**	
	**Hou et al**. **(****32****)**	**Blue–collar vs. white–collar**	**0.14 (*****p*** **=** **0.04)**		**coef**	
	Hou et al. (33)	Workers vs. white–collar	2.24 (1.12–4.48)		OR (95% CI)	
Education	**Marom et al**. **(****43****)**	**≤12 vs**. **>12 years**	**1.56 (0.97–2.52)**		**HR (95% CI)**	Strong
	**Hou et al**. **(****32****)**	**>12 vs**. **<** **9 years**		**0.41 (*****p*** **<** **0.001)**	**coef**	
	Hou et al. (33)	> 12 vs. <9 years		0.21 (0.09–0.50)	OR (95% CI)	
	Vuistiner et al. (49)	High education		1.26 (1.09–1.46)	HR (95% CI)	
Compensable status	**Marom et al**. **(****43****)**	**Recognized for benefit claim**	**0.88 (1.42–1.83)**		**HR (95% CI)**	Strong
	Ekegren et al. (30)	Private/Worksale vs. Medicare	0.33 (0.16–0.70)		OR (95% CI)	
	**Clay et al**. **(****27****)**	**No compensation**		**2.05 (1.20–3.49)**	**HR (95% CI)**	
Workload	**Marom et al**. **(****43****)**	**Workload/job control**	**0.58 (0.40–0.83)**		**HR (95% CI)**	Moderate
	Kirkeby et al. (39)	Forceful work	0.55 (0.30–0.99)		HR (95% CI)	
	Eisele et al. (29)	Low hand strain at work		2.33 (1.45–3.74)	HR (95% CI)	
	Ballabeni et al. (22)	High job strain	3.79 (1.54–9.31)		OR (95% CI)	
**Other factors (*****n*** **=** **14)**	**Marom et al**. **(****43****)**	**Legal counsel (yes vs. no)**	**0.53 (0.34–0.82)**		**HR (95% CI)**	Limited
	Neutel et al. (46)	Blame someone else for injury	1.70 (1.11–2.59		HR (95% CI)	Limited
	Eisele et al. (29)	Self employed vs. full time		1.77 (1.13–2.76)	HR (95% CI)	Limited
	Amick et al. (21)	Organizational policies		2.07 (1.18–3.62)	OR (95% CI)	Limited
	Kendrick et al. (37)	Threatening life event	0.27 (0.10–0.72)		OR (95% CI)	Limited
	**Murgatroyd et al**. **(****45****)**	**Full time vs. part–time**		**1.99 (1.26–3.14)**	**HR (95% CI)**	Limited
	Gabbe et al. (31)	Not at fault		0.92 (0.86–0.99)	RR (95% CI)	Limited
	**Luthi et al**. **(****42****)**	**Speak local language**		**0.67 (0.51–0.88)**	**OR (95% CI)**	Limited
	**Luthi et al**. **(****42****)**	**Restriction in integration**	**1.42 (1.24–1.61)**		**OR (95% CI)**	Limited
	Kong et al. (40)	Perception of social support		1.9 (1.2–3.0)	HR (95% CI)	Limited
	Kong et al. (40)	Family's attituted to RTW		4.0 (1.4–11)	HR (95% CI)	Limited
	Kong et al. (40)	Computer skill training (yes)		1.5(1.1–2.1)	HR (95% CI)	Limited
	Hou et al. (33)	Disturbance in daily life	2.10(1.02–4.30)		OR (95% CI)	Limited
	Hou et al. (33)	Married vs. others		0.50 (0.27–0.93)	OR (95% CI)	Limited

*RTW, Return to work; OR, odds ratio; RR, relative risk; Coef, coefficient; HR, Hazard ratio; CI, confidence interval*.

* *Barriers or facilitators for RTW were reported in positive or negative direction depending on the definition of outcome, the categories of interest, and the statistical reported*.

*Studies with a low risk of bias and high quality were bolded*.

### Biological Factors

#### Early Stage

Among the 7 biological factors, injury severity was the only factor supported by strong evidence, as it was reported in two studies with a low risk of bias and high quality (36, 47), in three studies with a moderate risk of bias (24, 25, 41), and in one study with a high risk of bias (35). There was limited evidence for the remaining 6 factors: age (25), gender (36), body mass index (41), initial need for surgery (25), level of pain (25), and disability level postinjury (35, 44) (Table 3).

#### Late Stage

Among the 20 biological factors, age, injury severity, and level of pain were supported by strong evidence. A significant relationship between older age and delayed RTW was found in six studies (23, 27, 30, 32, 33, 42), and three of them were rated as high quality and had a low risk of bias (27, 32, 42). A high level of injury severity as a barrier for RTW was identified in two studies with a low risk of bias and high quality (27, 45) and in six studies with a moderate risk of bias (23, 28, 30, 33, 40, 46). A significant relationship between a high level of pain and delayed RTW was reported in two studies with a low risk of bias and high quality (27, 43) and in two studies with a moderate risk of bias (34, 49). Inconsistent evidence was found for gender, as contradictory findings were reported in two studies (27, 46). There was limited evidence for the remaining 16 factors (Table 4).

### Psychological Factors

#### Early Stage

There was limited evidence for the six psychological factors: positive and negative effects (47), locus of control (47), prior depressive episode (41), quality-of-life psychological subscale (33), negative pain attitudes (25), and recovery belief (26) as predictors for RTW in the early stage (Table 3).

#### Late Stage

Among the 8 psychological factors, there was strong evidence for self-efficacy. The positive effect of self-efficacy on RTW has been reported in two studies with a low risk of bias and high quality (32, 43) and one study with a moderate risk of bias (33). Positive recovery expectations related to the better RTW outcome were supported by moderate evidence, as they were found in one study with a low risk of bias (45) and one study with a moderate risk of bias (49). Depressive or anxiety symptoms (33, 37) and perceived severity of injury (34, 49) were found in two studies with a moderate risk of bias, therefore resulting in limited evidence. There was limited evidence for intrusion thoughts (43), illness beliefs (23), avoidance (34), and mental health (27) (Table 4).

### Social Factors

#### Early Stage

Among the 20 social factors, there was limited evidence for educational level, as this factor was found in three studies with a moderate risk of bias (26, 40, 44). Of note, limited evidence was also found that blue-collar work was related to delayed RTW (25, 41). Limited evidence was found for other factors: type of contract (41), physical work (41), perception of social support (40), received compensation (26), injured at work (36), living in deprived areas (36), etc. (Table 3).

#### Late Stage

There was strong evidence for blue-collar work, educational level, and compensation status related to delayed RTW. Blue-collar work as a barrier for RTW was reported in two studies with a low risk of bias and high quality (32, 45) and two studies with a moderate risk of bias (33, 46). The positive effect of high educational level on RTW outcome was found in two studies with a low risk of bias and high quality (32, 43) and two studies with a moderate risk of bias (33, 49). Compensation status has been reported in two studies with a low risk of bias and high quality (27, 43) and one study with a moderate risk of bias (30). There was moderate evidence for physical workload, as this factor was reported in one study with a low risk of bias and high quality (43) and three studies with a moderate risk of bias (22, 29, 39). Other factors were supported by limited evidence (Table 4).

### Non-significant Factors for RTW

Numerous BPS factors showed no association with RTW in the early and late phases (Tables 5, 6, respectively).

**Table 5:** Non-significant factors for RTW in the early phase (≤ 6 months) postinjury.

**Factors**	**Authors**	**Categories of interest**	**Non-significant factors**	**Statistical reported**	**Levels of evidence**
**Biological factors**
Age	Izadi et al. (35)	Age (continuous)	−0.26 (−0.76 to 0.25)	coef (95% CI)	Limited
	Lilley et al. (41)	Age (55–64 vs. 18–24)	1.28 (0.86–1.91)	OR (95% CI)	
	Clay et al. (24)	41–62 vs. 18–40	2.13 (0.9–5.02)	OR (95% CI)	
Gender	Hou et al. (33)	Male vs. female	0.63 (0.36–1.08)	OR (95% CI)	Limited
	Lilley et al. (41)	Female vs. male	0.93 (0.72–1.20)	OR (95% CI)	
Pain	**Roesler et al**. **(****47****)**	**Pain (0–5 scale)**	**1.34 (p** **=** **0.27)**	**coef (** ***p*** **–value)**	Moderate
	Clay et al. (24)	Prior pain	0.97 (0.35–2.70)	OR (95% CI)	Limited
Others	Izadi et al. (35)	Disability post–injury	−0.01 (−0.32 to 0.29)	coef (95% CI)	Limited
	Clay et al. (24)	Pre–injury general health	2.33 (0.88–6.12)	OR (95% CI)	Limited
	Clay et al. (26)	General health at 2 weeks	0.95 (0.40–2.23)	OR (95% CI)	Limited
	Clay et al. (26)	Initial surgery required	0.59 (0.22–1.58)	OR (95% CI)	Limited
**Psychological factors**
Self–efficacy	Marom et al. (44)	Perception of self–efficacy	1.26 (0.83–1.93)	OR (95% CI)	Moderate
	**Roesler et al**. **(****47****)**	**The general self–efficacy scale**	**0.51 (*****p*** **=** **0.26)**	**coef (** ***p*** **–value)**	
Other factors	Marom et al. (44)	Intrusion	0.88 (0.60–1.28)	OR (95% CI)	Limited
	**Roesler et al**. **(****47****)**	**Psychological distress**	**2.55 (*****p*** **=** **0.096)**	**coef (** ***p*** **–value)**	Limited
	Clay et al. (24)	Recovery beliefs	1.92 (0.73–4.99)	OR (95% CI)	Limited
	Clay et al. (26)	Psychological distress	1.44 (0.55–3.72)	OR (95% CI)	Limited
**Social factors**
	Marom et al. (44)	Housing density	0.85 (0.37–1.91)	OR (95% CI)	Limited
	Marom et al. (44)	Level of occupation in Israel	1.00 (0.97–1.03)	OR (95% CI)	Limited
	Marom et al. (44)	Hand strength required	1.06 (0.58–1.92)	OR (95% CI)	Limited
	Marom et al. (44)	Repetitive hand motion	0.99 (0.69–1.43)	OR (95% CI)	Limited
	Marom et al. (44)	Lifting heavy loads	0.88 (0.60–1.30)	OR (95% CI)	Limited
	Marom et al. (44)	Workload/job control	0.87 (0.37–2.00)	OR (95% CI)	Limited
	Marom et al. (44)	Physical capability of the hand	0.99 (0.97–1.02)	OR (95% CI)	Limited
	Izadi et al. (35)	Work history	0.12 (−0.46 to 0.71)	coef (95% CI)	Limited
	Izadi et al. (35)	Cause of accident	−2.46 (−9.00 to 4.12)	coef (95% CI)	Limited
	**Roesler et al**. **(****47****)**	**Marital status**	**0.22 (*****p*** **=** **0.24)**	**coef (** ***p*** **–value)**	**Limited**
	**Kendrick et al**. **(****36****)**	**Road vs. home accidents**	**1.13 (0.54–2.35)**	**RR (0.95% CI)**	Limited
	Lilley et al. (41)	Sleep quantity and quality	0.79 (0.61–1.01)	OR (95% CI)	Limited
	Clay et al. (24)	Education	0.43 (0.14–1.29)	OR (95% CI)	Limited
	Clay et al. (24)	Work–related injury	1.21 (0.45–3.22)	OR (95% CI)	Limited
	Clay et al. (26)	Blue–collar work	0.53 (0.21–1.41)	OR (95% CI)	Limited
	Clay et al. (26)	Self–employment	1.21 (0.29–5.02)	OR (95% CI)	Limited

*OR, Odds ratio; RR, relative risk; Coef, coefficient; CI, confidence interval*.

*Studies with a low risk of bias and high quality were bolded*.

**Table 6:** Non-significant factors for RTW in the late phase (> 6 month) postinjury.

**Factors**	**Authors**	**Categories of interest**	**Non-significant factors**	**Statistical reported**	**Levels of evidence**
**Biological factors**
Age
	Kendrick et al. (37)	65–69 vs. 16–24	0.31 (0.06–1.68)	OR (95% CI)	Moderate
	Kirkeby et al. (39)	Age (continuous)	1.00 (0.98–1.02)	HR (95% CI)	
	**Murgatroyd et al**. **(****45****)**	**Age (continuous)**	**1.01 (0.99–1.02)**	**HR (95% CI)**	
**Gender**
	Kirkeby et al. (39)	Female vs. male	0.78 (0.47–2.33)	HR (95% CI)	Moderate
	Kendrick et al. (37)	Male vs. female	0.79 (0.45–1.38)	OR (95% CI)	
	**Murgatroyd et al**. **(****45****)**	**Male vs. female**	**0.96 (0.65–1.43)**	**HR (95% CI)**	
	Busse et al. (23)	Female vs. male	0.74 (0.33–1.69)	OR (95% CI)	
	Hou et al. (33)	Male vs. female	0.63 (0.36–1.09)	OR (95% CI)	
Smoking
	Kirkeby et al. (39)	Current vs. never smoker	0.80 (0.46–1.39)	HR (95% CI)	Limited
	Busse et al. (23)	Current vs. not currently smoking	0.68 (0.32–1.45)	OR (95% CI)	
Other factors	Marom et al. (44)	Ethnicity (Jews vs. Arabs)	1.22 (0.76–1.95)	HR (95% CI)	Limited
	Kirkeby et al. (39)	BMI (Obese vs. no obese)	0.79 (0.43–1.45)	HR (95% CI)	Limited
	Kirkeby et al. (39)	Injury of dominant hand	0.98 (0.64–1.51)	HR (95% CI)	Limited
	Iakova et al. (34)	General health at admission	1.4 (0.89–2.21)	OR (95% CI)	Limited
**Psychological factors**
	Marom et al. (43)	Avoidance	1.13 (0.88–1.40)	HR (95% CI)	Limited
	Kendrick et al. (37)	Crisis support scale	0.92 (0.88–0.97)	HR (95% CI)	Limited
	Iakova et al. (34)	Anxiety	0.94 (0.86–1.02)	HR (95% CI)	Limited
	Kong et al. (40)	Psychological counseling	3.8 (0.94–16)	HR (95% CI)	Limited
	Iakova et al. (34)	Expected outcome	1.14 (0.78–1.66)	OR (95% CI)	Limited
	Iakova et al. (34)	Intrusion	0.05 (0.6–1.84)	OR (95% CI)	Limited
	Iakova et al. (34)	Hyperarousal	1.32 (0.76–1.68)	OR (95% CI)	Limited
	Iakova et al. (34)	Mental score	1.15 (0.75–1.76)	OR (95% CI)	Limited
	Ballabeni et al. (22)	Job strain	1.78 (0.72–3.34)		Limited
**Social factors**
Education	Kirkeby et al. (39)	Education (low vs. high level)	1.22 (0.73–2.02)	HR (95% CI)	Moderate
	**Luthi et al**. **(****42****)**	**Higher education**	**0.79 (0.59–1.07)**	**OR (95% CI)**	
Other factors	**Marom et al**. **(****43****)**	**Partner working (Yes vs. no)**	**1.07 (0.65–1.75)**	**HR (95% CI)**	**Limited**
	**Marom et al**. **(****43****)**	**Lifting heavy loads**	**0.95 (0.81–1.12)**	**HR (95% CI)**	**Limited**
	Kirkeby et al. (39)	Repetitive work (>2.5hours/days)	0.75 (0.42–1.33	HR (95% CI)	Limited
	Kirkeby et al. (39)	Work with non–neutral postures	0.86 (0.48–1.53)	HR (95% CI)	Limited
	Vuistiner et al. (49)	Work contract	1.07 (0.88–1.30	HR (95% CI)	Limited
	**Luthi et al**. **(****42****)**	**Qualified work pre–injury**	**0.75 (0.56–1.01)**	**OR (95% CI)**	**Limited**
	**Luthi et al**. **(****42****)**	**Work–related injury**	**1.18 (0.93–1.3)**	**OR (95% CI)**	**Limited**
	Hou et al. (33)	Work compensation	2.31 (0.74–7.22)	OR (95% CI)	Limited

*OR, Odds- ratio; HR, Hazard ratio; CI, confidence interval; BMI, body mass index*.

*Studies with a low risk of bias and high-quality were bolded*.

#### Early Stage

There was moderate evidence for the level of pain (24, 47) and self-efficacy (44, 47) as non-significant factors for RTW in the early phase. These factors were identified in one study with a low risk of bias and one study with a moderate risk of bias. The evidence for age (24, 35, 41), sex (33, 41), and other biopsychosocial factors was limited, as they have only been identified in studies with moderate or high risk of bias.

#### Late Stage

There was moderate evidence for age (37, 39, 45), gender (23, 33, 37, 39, 45), and education (39, 42) as non-significant factors for RTW in the late phase. These factors were identified in one study with a low risk of bias and at least one study with a moderate risk of bias. There was limited evidence for other biopsychosocial factors (Table 6).

### Data Pooling

There was variability in the definition of RTW and its measurements across the included studies, and also in the reporting of prognostic factors (age, for example, as a dichotomous or continuous variable) and the types of statistical estimates (odds ratios or hazard ratios or risk ratios). All these barriers prevented data pooling; therefore, a meta-analysis could not be performed.

## Discussion

In this updated systematic review of 30 studies between 2010 and 2020, we were able to extract 33 significant factors for RTW in the early phase and 46 prognostic factors in the late phase. In agreement with the previous review of 15 studies published in 2010 (9), blue-collar work and educational level were supported by strong evidence. In addition, we found strong evidence for two new factors (age and level of pain) and moderate evidence for another two new factors (physical workload and recovery expectations). Importantly, injury severity, self-efficacy, and compensation status have been upgraded from moderate evidence (9) to strong evidence in this updated review. An earlier review in 2010 (9) did not have enough evidence to support the role of older age, injury severity, level of pain, self-efficacy, recovery expectations, and physical workload as the key barriers to RTW. The identification of these new factors was in accordance with evidence of predictors from the synthesis of 56 reviews on RTW in various conditions and injuries (7). Contrary to the earlier review in 2010 (9), the role of gender on RTW was inconsistent, as contradictory results were reported in the included studies (27, 46, 49).

The classification of predictors into early and late phase postinjury was one of the main differences between ours and the previous review (9). In the early phase following acute orthopedic trauma, strong evidence was found for injury severity only. A number of psychosocial factors, including self-efficacy, recovery expectations, blue-collar work, and physical demand, had limited evidence in the early phase but became more evident in the late phase postinjury. This is likely because while most of the injured workers return to work in a straightforward pathway soon after the acute phase, a proportion of patients might turn into the chronic work disability process. In these patients, psychosocial problems might play an important role in the late phase and interact with other factors as the key barriers to RTW. Another explanation was that the longer the duration of follow-up, the greater the likelihood of recognizing the significant effects of psychosocial problems on RTW.

Another difference was that we assessed both the significant and non-significant effects of all reported predictors. For example, increasing age showed no relationship with RTW in three studies (37, 39, 45), but it significantly predicted RTW in the other six studies (23, 27, 30, 32, 33, 42). The difference might be due to selection bias. For studies demonstrating the impact of age, over half of the injured population were blue-collar or immigrant workers (32, 33, 42). In other words, older blue-collar workers are likely to have more difficulty reentering the labor market than young white-collar workers. The predictive validity of age, therefore, must be interpreted in conjunction with the BPS context, for example, with the occupation. Careful interpretation is also needed for educational level, as this factor has shown no significant effect on RTW in two studies (39, 42), whereas other studies have demonstrated a significant correlation (32, 33, 43, 49). The discrepancy between these studies might be due to the different categories of predictors and duration of follow-up.

Understanding the levels of evidence of prognostic factors for postinjury employment helps to identify patients at high risk for poor RTW outcomes and to improve guidance for RTW. According to our results, injury severity was recognized as one of the principal barriers to RTW in workers; therefore, public health and work environments should pay attention to serious injury prevention, as the majority of accidents are preventable (51). Whereas, the prevalence of workplace injuries seemed to be reduced from 2010 to 2018 in Europe thanks to the European strategic framework on health and safety at work (52), the prevalence of non-work injuries (domestic, road, and leisure time injuries) remained high. Public health should take action to improve road safety legislation, road infrastructure, and first trauma care as core measures for reducing the burden of road accidents. Prevention campaigns for other injury causes, especially sports injuries, are necessary as well.

Older injured workers might need special promoting policies to enhance RTW, especially blue-collar workers, including adapting work accommodations. In addition, providing access to interdisciplinary treatment for pain is also of importance. Health professionals, however, should remain mindful that non-biological factors such as self-efficacy, recovery expectations, blue-collar work, and physical workload also contribute significantly to the RTW outcome. Adapting physical workload, for example, offering lighter or modified or graded work exposure or performing onsite work evaluation, may help to increase the success of RTW. Our findings support the “seven principles for successful RTW” previously established for enhancing RTW in musculoskeletal or pain-related conditions (53–55). It is also suggested that self-efficacy and recovery expectations are relevant factors that need to be screened in workers as early as possible after injury. Higher self-efficacy and recovery expectations can be obtained by support from leaders and coworkers to promote RTW (56). Surprisingly, work-related factors such as support from leaders and coworkers were not reported in the included studies. Hopefully, future researchers will strive to improve reporting on this factor.

Worker compensation has been supported in our updated review by strong evidence. However, workers' compensation systems are different from country to country, and the interpretation of this finding needs to be cautious. For example, most European countries compensate workers for both professional and non-professional accidents, and coverage is provided regardless of fault. Another important issue of concern in workers' compensation is the source of insurance. In many European countries, public organizations control workers' compensation insurance policies, whereas in some countries (for example, Australia, UK), insurance can be provided either directly through the employer or through a private insurance provider. The negative impact of compensation on RTW was reported in countries where private insurance companies were involved in the sick pay scheme (27, 30, 43). Workers' compensation status was not related to RTW in Taiwan (33), where the public labor system pays injured workers their lost wages for 2 years postinjury.

It should be noted that other factors (depression, psychological disorders, social support, vocational training, etc.) were rated as limited evidence in the early and late phases because the number of high-quality studies required for qualification has not been reached. They should not be interpreted as factors of limited importance. These factors may need to be addressed in further high-quality studies to determine whether they are relevant in the RTW process after orthopedic trauma. Many of these factors are potentially amenable to intervention. For example, psychological disorders may benefit from psychological care or cognitive behavioral therapy; computer skills can be acquired from vocational training.

From the methodological point of view, the inclusion and exclusion criteria were quite similar between ours and the previous review (inclusion of longitudinal studies of patients sustaining orthopedic trauma, and exclusion of studies that did not recognize the multifactorial nature of RTW) (9). Unlike the specific criteria applied in the previous review (9), we used the QUIPS, a recently validated tool, to assess the risk of bias of all included studies. For one study presenting the predictive model (42), the risk of bias was also assessed by PROBAST, which resulted in the same level of risk of bias. We found that study attribution and confounding were the most common types of bias risk. Some contributing studies did not clearly describe the rates of loss to follow-up or the potential impact of subjects lost to follow-up (33, 35, 39), which are important elements affecting study attribution. The methods for missing data have not been appropriately handled in some studies (31, 34, 50), resulting in a source of reduced statistical power. Apart from the assessment of the risk of bias, the quality of all included cohorts was also evaluated by the NOS. It was demonstrated that all seven studies with a low risk of bias were rated as having high quality. Two studies with a high risk of bias were rated as having medium quality. The remaining 11 studies of moderate risk of bias were rated as having high quality in nine studies and medium quality in two studies.

There has been a lack of consistency in the definition of the RTW outcome, as it can be defined as sustained RTW, fitness to work, or simply yes/no status. Likewise, the prognostic factors were measured in different ways (even for age: continuously and dichotomously) at different time points after the traumatic event. It should be noted that until present, most of the included studies were predicting factor studies that focus only on the associative relationship between prognostic factors and RTW outcome. Studies evaluating the predictive model's performance, for example, the external validation of the model, remain limited.

### Strengths and Limitations

The strengths of this review are as follows: First, this review was conducted following the PRISMA recommendations for systematic reviews (15). The high number of included studies (*n* = 30) and the clear predefined inclusion criteria (cohort studies with longitudinal data on RTW, and only results from multiple regression models were considered) allowed us to establish robust conclusions about the validity of predictors. In addition, to provide an objective evaluation, all enrolled studies were independently assessed by the two reviewers. The quality and risk of bias were evaluated by validated tools recommended by the Cochrane Methods Prognosis group (57). The levels of evidence of biopsychosocial factors were made based on the high quality and low risk of bias studies.

This review also has some limitations. First, we could not regroup relevant prognostic factors in a meta-analysis due to variability in the definitions and measurements of the outcomes and predictors. Second, the quality and risk of bias tools involved a degree of subjectivity; however, this was solved by careful discussion. Third, the exclusion of studies of non-orthopedic injuries (for example, traumatic brain injury or internal organ injury) prevents the generalization of findings to other injuries. Last, to avoid the overlooking of evidence, the inclusion criteria (prospective studies, sample size >80 participants) may have resulted in the exclusion of potentially relevant studies.

### Suggestions for Improvement of Research in This Area

The definition of RTW remains inconsistent, and further consensus on its definition is needed. Recently, some RTW questionnaires have been developed to measure the different aspects of RTW in patients with work-related injury (58, 59). These new questionnaires might be used in the future to assess the multiple dimensions of RTW outcomes. Likewise, some prognostic factors need to be measured or categorized uniformly (for example, age as a continuous variable) to ensure that data pooling can be performed. Moreover, the majority of predicting factor studies were at the developing stage, without validating performance in new patients. We suppose that validating and studying the clinical impact of a prediction model RTW rather than the usual reporting of predictive values could help to guide an efficient strategy. To improve the quality of studies and reduce the risk of bias, it is necessary to report the rate of loss to follow-up and to provide appropriate statistical methods for missing data in the study. There is also a gap in the literature regarding the effects of analgesic prescriptions (especially opioids) and work-related factors such as support from leaders and coworkers on RTW after acute orthopedic trauma. It would be useful to conduct further research on these factors to acknowledge their roles in the RTW process. None of the included articles in this review originated from middle- or low-income countries, and there is a need to know the situation in these countries as well.

## Conclusion

In this updated systematic review of 30 studies between 2010 and 2020, injury severity was identified as a key barrier for RTW in the early and late phases postorthopedic injury. In the late phase postinjury, there was strong evidence for age, level of pain, self-efficacy, educational level, blue-collar work, and compensation status and moderate evidence for recovery expectations and physical workload as prognostic factors for RTW. Other factors were classified as having limited or inconsistent evidence, and further high-quality studies are needed to understand their impacts. The results from this current update might help in developing effective intervention strategies for RTW and in guiding future research in the field.

## Data Availability Statement

The original contributions presented in the study are included in the article/supplementary material, further inquiries can be directed to the corresponding author/s.

## Author Contributions

HD: involved in conceptualization, data curation, formal analysis, validation, visualization, writing original draft, and writing, reviewing, and editing. AG: prepared conceptualization, data curation, formal analysis, validation, visualization, writing original draft, and writing, reviewing, and editing. RH: contributed in conceptualization, data curation, formal analysis, project administration, supervision, validation, visualization, writing original draft, and writing, reviewing, editing. BL: carried out conceptualization, methodology, project administration, supervision, validation, visualization, writing, reviewing, and editing. FL: carried out conceptualization, methodology, data curation, project administration, supervision, validation, visualization, writing original draft, writing, reviewing, and editing. All authors contributed to the article and approved the submitted version.

## Conflict of Interest

The authors declare that the research was conducted in the absence of any commercial or financial relationships that could be construed as a potential conflict of interest.

## Publisher's Note

All claims expressed in this article are solely those of the authors and do not necessarily represent those of their affiliated organizations, or those of the publisher, the editors and the reviewers. Any product that may be evaluated in this article, or claim that may be made by its manufacturer, is not guaranteed or endorsed by the publisher.

## Abbreviations

BPS: Biopsychosocial
NOS: Newcastle–Ottawa Scale
PRISMA: Preferred Reporting Items for Systematic reviews and Meta-Analysis
PROBAST: Prediction model risk of bias assessment tool
QUIPS: Quality in Prognosis Studies
RTW: Return to work.
